# Integrating Genetic Variants and Expression Profiles of Pharmacogenes to Investigate Resistance to Antidepressant Treatment

**DOI:** 10.3390/medicina62050965

**Published:** 2026-05-15

**Authors:** Claudia Pisanu, Alessio Squassina, Júlia Perera-Bel, Rosana Carvalho Silva, Lisa Buson, Anna Martinez Sires, Marco Bortolomasi, Valentina Menesello, Giulia Perusi, Bernardo Carpiniello, Ewa Ferensztajn-Rochowiak, Filip Rybakowski, Ferran Sanz, Mirko Manchia, Marie Claude Potier, Mara Dierssen, PROMPT Study Group, Bernhard T. Baune, Massimo Gennarelli, Alessandra Minelli

**Affiliations:** 1 Section of Neuroscience and Clinical Pharmacology, Department of Biomedical Sciences, University of Cagliari, 09042 Monserrato, Italy; claudia.pisanu@unica.it; 2 Hospital del Mar Medical Research Institute (HMRIB), 08003 Barcelona, Spain; jperera@researchmar.net (J.P.-B.); annams31@gmail.com (A.M.S.); ferran.sanz@upf.edu (F.S.); 3 Department of Molecular and Translational Medicine, University of Brescia, 25121 Brescia, Italy; r.carvalhosilva@unibs.it (R.C.S.); valentina.menesello@gmail.com (V.M.); giuliaperusi@gmail.com (G.P.); gennarelli@fatebenefratelli.eu (M.G.); 4 Genetics Unit, IRCCS Istituto Centro San Giovanni di Dio Fatebenefratelli, 25125 Brescia, Italy; lisa.buson@gmail.com; 5 Psychiatric Hospital “Villa Santa Chiara”, 37142 Verona, Italy; marcobortolomasi.vr@gmail.com; 6 Section of Psychiatry, Department of Medical Sciences and Public Health, University of Cagliari, 09124 Cagliari, Italy; bcarpini@iol.it (B.C.); or mirko.manchia@dal.ca (M.M.); 7 Department of Adult Psychiatry, Poznan University of Medical Sciences, 61-701 Poznan, Poland; ferensztajnewa@gmail.com (E.F.-R.); filrybak@yahoo.com (F.R.); 8 Department of Medicine and Life Sciences, Universitat Pompeu Fabra, 08005 Barcelona, Spain; 9 Department of Pharmacology, Dalhousie University, Halifax, NS B3H 4R2, Canada; 10 Paris Brain Institute (ICM), National Centre for Scientific Research (CNRS), 75794 Paris, France; marie-claude.potier@upmc.fr; 11 Centre for Genomic Regulation (CRG), The Barcelona Institute for Science and Technology (BIST), 08036 Barcelona, Spain; mara.dierssen@crg.eu; 12 Biomedical Research Networking Center for Rare Diseases (CIBERER), 08003 Barcelona, Spain; 13 Department of Psychiatry, University of Münster, 48149 Münster, Germany; bernhard.baune@uni-muenster.de; 14 Florey Institute of Neuroscience and Mental Health, Parkville, VIC 3052, Australia; 15 Department of Psychiatry, University of Melbourne, Parkville, VIC 3010, Australia

**Keywords:** pharmacogenetics, precision psychiatry, CYP2D6, CYP2C19, antidepressants, treatment-resistant depression, mRNA, miRNA

## Abstract

*Background and Objectives*: Treatment-resistant depression (TRD) is a major clinical challenge in the management of major depressive disorder (MDD). While pharmacogenetics has been suggested to be clinically useful in guiding antidepressant treatment, few studies have explored if and how pharmacogenes can be involved in TRD pathophysiology and its clinical outcomes. *Material amd Methods*: We explored the role of differences in metabolizer phenotypes, gene expression levels, and microRNAs of three key pharmacogenes (CYP2D6, CYP2C19, CYP2B6) in TRD pathophysiology and antidepressant response in a cohort of 300 patients with MDD from the PROMPT consortium. *Results*: *CYP2D6* phenotype distribution did not differ significantly between TRD and non-TRD groups, but mRNA expression was significantly upregulated in TRD. Hsa-miR-26b-5p, a microRNA predicted to regulate *CYP2D6*, was significantly downregulated in TRD. For CYP2C19, intermediate metabolizers (IMs) were underrepresented in TRD versus non-TRD (IMs vs. normal metabolizers (NMs): χ^2^ = 6.07, *p* = 0.019). microRNA hsa-let-7d-5p and hsa-miR-27a-3p, predicted to regulate CYP2C19, were significantly downregulated in TRD. No significant differences were found for CYP2B6. *Conclusions*: This study contributes valuable insights to the PROMPT project on how pharmacokinetic gene variants and their expression and regulatory mechanisms may influence antidepressant response and resistance in MDD.

## 1. Introduction

Major depressive disorder (MDD) is the most common and disabling mental illness and associated with significant functional impairment, reduced quality of life, and socioeconomic impact. It is characterized by persistent low mood, loss of interest in previously enjoyable activities, and is often accompanied by low self-esteem, reduced energy, and feelings of sadness or hopelessness without a clear cause [1]. Diagnosing and managing MDD can be challenging due to heterogeneity in its clinical presentation, variable clinical course, and inconsistent treatment response. Pharmacological treatment is typically the first-line approach, and involves the use of different classes of antidepressants alone or in combination. However, only about 30% of patients achieve remission with the first pharmacological trial, and approximately 15%–30% develop treatment-resistant depression (TRD) [2,3]. Treatment failure is further complicated by side effects and tolerability issues, which lead many patients to discontinue medication [4,5]. Finding an effective treatment often requires multiple trials, with the chance of success decreasing with each unsuccessful attempt, contributing to prolonged symptoms, worse long-term prognosis, and increased personal and societal costs [6].

This high variability in antidepressant effectiveness is influenced by both genetic and non-genetic factors, and while the role of genetic variations has yet to be elucidated, in the last two decades the pharmacogenetics of response to antidepressants has been increasingly investigated. Pharmacogenetics can help improve our understanding of how patient-specific genetically driven variability in the pharmacokinetics and pharmacokinetics of drugs can influence efficacy and safety, leading to personalized treatments and reducing the risk of treatment discontinuation, especially in patients with TRD. Despite the role of genetics being consolidated over the years, few genes involved in drug metabolism have been reported to significantly impact treatment outcomes and adverse reactions in antidepressant treatment [7]. Indeed, it has been suggested that only a small group of genes, namely those coding for cytochromes (CYPs)—CYP2D6, CYP2C19, and CYP2B6—that are involved in antidepressant metabolism may influence the effectiveness and safety of these medications, but their association with TRD has been inadequately studied [7,8].

Traditionally, CYP-based pharmacogenetics is based on the “diplotype-inferred metabolizer phenotype,” where the presence of specific genetic variants is used to predict enzyme activity and guide dosing. As such, only a limited number of exploratory studies incorporated into pharmacogenetic tools other molecular measures possibly impacting CYP-metabolizing properties, such as their gene expression or epigenetic mechanisms involved in modulating their transcription [7]. Differences in the expression and splicing of genes encoding drug-metabolizing enzymes, transporters, and targets, such as receptors and ion channels, have been associated with interindividual variability in optimal drug dosing, therapeutic effectiveness, and side effects [9]. Nevertheless, this has only been limitedly explored in relation to antidepressant treatment in MDD. Recent findings suggest that the transcriptional regulation of pharmacogenes, including variability in mRNA expression and modulation by microRNAs (miRNAs), may significantly influence drug metabolism independently of genetic diplotypes [9,10,11]. This suggests that the expression of pharmacogenes, such as CYP2D6 and CYP2C19, can vary within the same genotype-predicted metabolizer groups, potentially altering antidepressant efficacy. Moreover, it has been shown that miRNAs may affect both the pharmacokinetics and dynamics of antidepressants and play a role in the pathophysiology of depression itself [12]. Nonetheless, studies integrating genotyping and expression data in the pharmacogenetics of antidepressants remain scarce, especially in patients with TRD.

Here, we explored the hypothesis that CYP-related molecular measures other than genetically inferred metabolizing phenotypes and/or the interaction between them might be associated with TRD and remission to antidepressants.

To this end, we performed a cross-sectional, retrospective study in a cohort of MDD patients stratified into TRD and non-TRD and remitters or non-remitters after treatment with antidepressants collected within the PROMPT consortium [13]. Genes explored included those with the highest level of evidence of clinical utility for antidepressants reported in ClinPGX (CYP2D6, CYP2C19, and CYP2B6). Specifically, our study investigated: (1) whether patients with TRD show a different distribution of CYP diplotypes and clinical features compared to non-TRD; (2) whether the blood expression of CYP genes varies between TRD and non-TRD groups; (3) and whether the expression levels of miRNAs regulating these pharmacogenes significantly differ between TRD and non-TRD patients. By using an integrated approach, our study aimed to provide more insight into the involvement of CYP genes and the epigenetic mechanisms regulating their expression in TRD and in response to antidepressants.

## 2. Materials and Methods

### 2.1. Study Participants and Clinical Assessment

This study included 300 patients with MDD from a retrospective cohort (IRCCS Fatebenefratelli, Brescia, Italy) participating in the phase I PROMPT project (Toward PrecisiOn Medicine for the Prediction of Treatment response in major depressive disorder through stratification of combined clinical and -omics signatures [13]). The diagnostic criterion for inclusion was a diagnosis of moderate to severe MDD according to the Diagnostic and Statistical Manual of Mental Disorders, Fourth Edition (DSM-IV) classification system. The diagnosis of MDD was confirmed for all participants using the Italian version of the SCID-I diagnostic scale according to DSM-IV criteria. Personality disorders were diagnosed based on clinical symptom evaluation consistent with DSM-IV standards. Exclusion criteria included: (a) a lifetime history of schizophrenic, schizoaffective, or bipolar disorder; (b) primary diagnosis of personality disorder, substance abuse, alcohol abuse, or dependence, obsessive–compulsive disorder (OCD), or posttraumatic stress disorder (PTSD); (c) comorbidity with eating disorders; (d) comorbidity with alcohol and substance dependence; (e) intellectual disability and cognitive impairment; (f) neurological disorders such as Parkinson’s disease, multiple sclerosis, Alzheimer’s disease and other dementias, epilepsy, strokes, brain tumors, and traumatic conditions of the nervous system; (g) comorbidity with other severe medical illnesses and severe autoimmune diseases such as cancers, Crohn’s disease, rheumatoid arthritis, scleroderma, psoriasis, myasthenia gravis, Sjögren’s syndrome, and systemic lupus erythematosus; and (h) pregnancy.

Half of the patients (150) were classified as having TRD and the other 150 classified as non-TRD. Based on clinical judgment by the treating psychiatrists, TRD was defined as a failure of treatment to produce response or remission for patients after two or more treatment attempts of adequate and recommended dose and duration [14]. Most TRD patients had a long-standing history of the condition and had undergone ECT or other intensive treatments, including drug combinations, augmentation with antipsychotics and/or mood stabilizers, intensive psychotherapy, repetitive transcranial magnetic stimulation (rTMS), and sleep deprivation. MDD patients were classified as non-TRD when they achieved response or remission in terms of a reduction in symptomatology with the current antidepressant treatment attempt of adequate dose and duration. All non-TRD patients were either at their first MDD episode or had other episode/s in which they responded to the treatment received or had only one failure in their pharmacological anamnesis history.

Baseline symptoms evaluations were made using Montgomery–Åsberg Depression Rating Scale (MADRS) at the presentation of the patients to psychiatric services or hospital, in concomitance with blood collection.

For a subgroup of patients (*n* = 228), response to treatment in the current episode was available as assessed with the MADRS after 8 weeks. These data from naturalistic observational cohorts in real-world conditions allowed us to classify TRD patients as responders and non-responders and TRD and non-TRD patients as remitters and non-remitters. Response was defined as a ≥50% decrease in MADRS score after 8 weeks of treatments, whereas remission was defined as a score of ≤9 in MADRS score.

For all patients, information such as age, sex body mass index (BMI), smoking, age of onset, severity, and psychiatric comorbidities were collected (Table 1).

Clinical features of the sample stratified based on remission status are reported in Supplementary Table S1.

The study was approved by the local ethics committee (CEIOC IRCCS Istituto Centro San Giovanni di Dio Fatebenefratelli, Brescia, Italy; registration 62/2021, approved on 5 November 2021) in agreement with coordinator ethics committee approval (Ethik-Kommission Westfalen-Lippe der Ärztekammer Westfalen-Lippe, Münster, Germany; registration 2021-103-f-S, approved 26 February 2021). Considering this work reports findings from a retrospective study (PROMPT phase I) involving patients recruited within previous projects and for whom biological samples were made available for the PROMPT project, informed consent was waived.

### 2.2. RNA Sequencing and Genotyping

Peripheral venous blood samples were collected after an overnight fast between 8 and 9 a.m., in EDTA tubes for DNA extraction and in PAXGene Blood RNA Tubes (Qiagen, Hilden, Germany) for RNA extraction and mRNA/miRNA sequencing. For mRNAs, libraries were prepared using the Illumina Stranded Total RNA Prep with Ribo-Zero Plus kit (Illumina, San Diego, CA, USA) and sequenced on an Illumina NovaSeq 6000 platform (2 × 30 million 100-base pair reads per sample) [15], while for miRNAs, libraries were prepared using the NEBNext^®^ Small RNA Library Prep Set for Illumina^®^ kit (San Diego, CA, USA) (ref. E7330) and sequenced 1× 75 + 8 bp on an Illumina’s NextSeq500 (San Diego, CA, USA) [15]. For mRNA and miRNA sequencing analyses, quality control and statistical analyses procedures included RNA-seq preprocessing conducted using STAR 2.7.8a [16] and featureCounts from Subread v2.0.3 [17], normalization of gene expression counts in edgeR v.3.40.2, and removal of batch effects using the limma R package 3.66 [15]. After quality control, mRNA analyses included 293 participants and 21,564 genes, while miRNA analyses included 299 participants and 1045 miRNAs.

For genotyping, genomic DNA was extracted from whole blood using the QIAamp DNA Blood Midi Kit (Qiagen), while the quantity and the quality of the DNA were evaluated through spectrophotometric analysis (NanoDrop 2000, Thermo Scientific, Waltham, MA, USA). Twenty-nine genetic variants in CYP2D6 (*n* = 15), CYP2C19 (*n* = 10), and CYP2B6 (*n* = 4), selected based on the list published by the ClinPGX Association for Molecular Pathology (AMP) PGx Working Group (https://www.ClinPGX.org/ampAllelesToTest, accesed on 8 January 2026) were genotyped with customized TaqMan OpenArray plates (Waltham, MA, USA) on QuantStudio 12K Flex Real-Time PCR System (Applied Biosystems, Foster City, CA, USA) as per the manufacturer’s instructions. Copy number variation (CNV) of the CYP2D6 gene was evaluated using the TaqMan Copy Number Assay mix specific for CYP2D6 exon 9 (Applied Biosystems, Foster City, CA, USA) (Assay ID: Hs00010001_cn) as per the manufacturer’s instructions. Using the AlleleTyper Software (v 1.0) (Life Technologies, Carlsbad, CA, USA), single-nucleotide polymorphism (SNP) genotyping results were integrated with the copy number information for the CYP2D6 gene. If determination was uncertain, the diplotype with the higher allele frequency in the European population was selected. Genotype-inferred phenotyping was carried out as per CPIC guidelines (https://cpicpgx.orgm accesed on 8 January 2026). Details about haplotype-inferred phenotypes are reported in Supplementary Table S2.

### 2.3. Statistical Analysis

The distribution of continuous variables was tested with the Kolmogorov–Smirnov test. Differences in sociodemographic and clinical variables among the studied groups were tested with the Mann–Whitney U test or Pearson’s chi-squared test. We tested associations between the metabolizer phenotypes of pharmacogenes and three clinical outcomes: (1) TRD vs. non TRD, (2) extreme phenotypes of treatment response (TRD non-remitters vs. non-TRD remitters), and (3) remission vs. non-remission in patients stratified for TRD. Associations between the metabolizer phenotypes and the three clinical outcomes were tested with Pearson’s chi-squared test. Associations between the status of being a carrier of one or more altered function phenotypes and clinical outcomes were also tested. To identify potential confounders, associations between metabolizer phenotypes and socioeconomic or clinical features were tested with the Kruskal–Wallis test or Pearson’s chi squared test (Supplementary Table S1). In cases of significant association between being a carrier of altered-function phenotypes and clinical outcomes, a binary logistic regression model adjusted for confounders associated with pharmacogenes was also constructed. We also conducted analyses with TRD or extreme phenotypes as the outcome, restricted to the subsample of patients treated with antidepressants for which clinical annotations at level 1a or 2a are available in ClinPGX for CYP2D6 or CYP2C19. No analyses were conducted for CYP2B6, since only 29 participants were treated with sertraline, the only antidepressant for which a clinical annotation is available for this enzyme or with remission as the outcome, and therefore an insufficient number of patients who would have been included. A total of 139 patients (97 with TRD and 42 without TRD) were treated with antidepressants for which clinical annotations at level 1a or 2a are available for CYP2D6 (amitriptyline, clomipramine, fluvoxamine, mirtazapine, nortriptyline, paroxetine, venlafaxine, and vortioxetine), while 125 (56 with TRD and 69 without TRD) were treated with antidepressants for which clinical annotations at level 1a or 2a are available for CYP2C19 (amitriptyline, citalopram, clomipramine, escitalopram, and sertraline). For analyses on the metabolizer phenotypes of the three pharmacogenes, a *p*-value adjusted based on Bonferroni correction for three tests was significant (i.e., 0.05/3 = 0.016).

For mRNA and miRNA analyses, principal component analysis and variance partitioning analysis were used to assess the variance of the data and select relevant variables among batch, age, sex, BMI, age of onset, and MADRS [15]. Since age, sex, and batch were the main sources of variance, the batch effect was corrected with the removeBatchEffect function from the limma package, while differential expression analysis adjusted for age and sex was conducted using the limma-voom pipeline (limma version 3.54.1). *p*-values were adjusted for multiple testing using the Benjamini–Hochberg method, with a threshold of false-discovery rate (FDR) < 0.05. For the present study, only log2-fold-change (log-FC), *p*-values, and FDR adjusted (adj) *p*-values of selected genes (CYP2D6, CYP2C19, and CYP2B6) and regulating-miRNAs were extracted. However, mRNA association statistics were available only for CYP2D6, while CYP2C19 and CYP2B6 were too lowly expressed to be analyzed. Experimentally supported and predicted miRNAs regulating the three pharmacogenes were identified with TarBase v. 9.0 [18]. We identified one miRNA for CYP2D6 (hsa-miR-26b-5p), seven for CYP2C19 (hsa-miR-30d-3p, hsa-let-7d-5p, hsa-miR-139-5p, hsa-miR-210-3p, hsa-miR-27a-3p, hsa-miR-3662, and hsa-miR-423-5p), and none for CYP2B6. Analyses were performed with SPSS (v. 29).

## 3. Results

### 3.1. Associations Between CYP2D6 and TRD

CYP2D6 showed a significant association with comorbidity with anxiety disorders in the whole sample (Supplementary Table S3). Frequency of CYP2D6 phenotypes did not show significant differences between patients with TRD and those without TRD or when considering extreme phenotypes of response (Table 2 and Supplementary Table S4).

Similarly, no difference was observed when restricting the analysis to patients treated with antidepressants for which clinical annotations with a high level of evidence for CYP2D6 are available on ClinPGX (Supplementary Table S5). Conversely, CYP2D6 mRNA was upregulated in patients with TRD compared to those without TRD (logFC = 0.35, t = 4.54, *p* = 8.3 × 10^−6^, adj *p* = 0.0002; Table 3).

In the extreme-phenotype comparison, CYP2D6 showed nominal significance, with the same direction of effect (logFC = 0.29, t = 2.47, *p* = 0.014, adj *p* = 0.11; Table 3). hsa-miR-26b-5p was the only miRNA predicted to regulate CYP2D6 levels based on TarBase and was significantly downregulated in patients with TRD compared to those without TRD (logFC = −0.62, t = −5.27, *p* = 2.6 × 10^−7^, adj *p* = 2.5 × 10^−5^; Table 3). Consistent results were observed in the extreme-phenotype comparison, with hsa-miR-26b-5p found to be nominally downregulated in TRD non-remitters patients compared with non-TRD remitters (logFC = −0.44, t = −2.33, *p* = 0.02, adj *p* = 0.21; Table 3).

### 3.2. Associations Between CYP2C19 and TRD

CYP2C19 showed a significant association with age at recruitment in the whole sample and in patients with TRD (Supplementary Table S1). CYP2C19 phenotypes showed a nominally significant association with TRD (χ^2^= 9.64, *p* = 0.047, Table 2 and Supplementary Table S6) in the whole sample, but not when limiting the analysis to patients treated with antidepressants with clinical annotations for CYP2C19 reported in ClinPGX (Supplementary Table S7). Intermediate metabolizers (IMs) (TRD: 20%, non-TRD: 32%) were significantly less represented in TRD than in non-TRD patients (IMs vs. normal metabolizers (NMs): χ^2^ = 6.07, *p* = 0.019). Similar results were also found when grouping the impaired function phenotypes (poor metabolizers (PMs) + IMs vs NMs: χ^2^= 9.48, *p* = 0.004, Supplementary Table S6). The association remained nominally significant in a binary logistic regression model adjusted for age at recruitment (IMs vs. NMs: odds ratio (OR) = 0.54, standard error = 0.31, *p* = 0.043; PMs + IMs vs. NMs: OR = 0.72, standard error = 0.15, *p* = 0.024).

In the comparison of extreme phenotypes of response, CYP2C19 phenotypes showed a significant association with non-remission in TRD (χ^2^= 13.92, *p* = 0.008, Table 2). In detail, IMs were less represented (IMs: 19.3% vs. 44.0%) in TRD non-remitters than non-TRD remitters (IMs vs. NMs: χ^2^ = 9.84, *p* = 0.002; PMs + IMs vs. NMs: χ^2^= 9.48, *p* = 0.004, Supplementary Table S6). The association remained significant in a binary logistic regression model adjusted for age at recruitment (IMs vs. NMs: OR = 0.28, standard error = 0.49, *p* = 0.008; PMs + IMs vs. NMs: OR = 0.53, standard error = 0.23, *p* = 0.006).

mRNA counts for CYP2C19 were not available due to low expression. Of seven miRNAs found to regulate CYP2C19 levels based on TarBase, data were available for six miRNAs, two of which showed a significant association with TRD. Specifically, hsa-let-7d-5p (logFC = −0.31, t = −4.18, *p* = 3.9 × 10^−5^, adj *p* = 0.002) and hsa-miR-27a-3p (logFC = −0.38, t = −4.35, *p* = 1.9 × 10^−5^, adj *p* = 0.0008) were significantly downregulated in patients with TRD compared with patients without TRD (Table 3). Two other miRNAs, hsa-miR-139-5p and hsa-miR-210-3p, were nominally down- and upregulated, respectively, in patients with TRD compared with non-TRD (Table 3). Among these, only hsa-miR-27a-3p showed a nominal association in the extreme-phenotype analysis (logFC = −0.44, t = −3.14, *p* = 0.002, adj *p* = 0.07).

### 3.3. Associations Between CYP2B6 and TRD

CYP2B6 showed a significant association with smoking in patients with non-TRD and with recurrence in patients with MDD (Supplementary Table S2). The frequency of CYP2B6 metabolizer phenotypes was not associated with TRD (Table 2). While we identified a significant association between CYP2B6 metabolizer phenotypes and the extreme phenotype of response (Table 2), we found no significant association when evaluating the status of being a carrier of one or more specific metabolizer phenotypes compared to NMs (Supplementary Table S8). The statistics for the mRNA of this gene were not available due to low expression, and no miRNA was identified to regulate CYP2B6 levels on TarBase.

### 3.4. Associations Between Pharmacogenes and Remission

We observed no significant association between CYP2D6 phenotypes and remission in patients with or without TRD (Table 4).

Analyses on specific metabolizer phenotypes showed a trend for higher frequency of CYP2D6 ultrarapid metabolizers (UMs) in remitters compared with non-remitters in patients with TRD (χ^2^= 4.76, *p* = 0.045), but not in patients without TRD (χ^2^= 0.02, *p* = 1.00, Supplementary Table S9). In addition, lower levels of CYP2D6 were nominally associated with non-remission in patients with TRD (logFC = −0.25, t = −2.11, *p* = 0.04, adj *p* = 0.39), but not in patients without TRD (logFC = −0.06, t = −0.45, *p* = 0.65, adj *p* = 0.99). Consistently, hsa-miR-26b-5p was nominally upregulated in non-remitters compared with remitters in patients with TRD (logFC = 0.40, t = 2.08, *p* = 0.04, adj *p* = 0.51), but not in those without TRD (logFC = −0.05, t = −0.26, *p* = 0.80, adj *p* = 0.99).

Frequency of CYP2C19 phenotypes showed a nominally significant trend of association with remission among patients without TRD, but not in patients with TRD (Table 4). Analyses on specific metabolizer phenotypes showed a nominally significant higher frequency of reduced function phenotypes (IMs vs. NMs: χ^2^ = 5.48, *p* = 0.036; PMs + IMs vs. NMs: χ^2^= 5.99, *p* = 0.022) in remitters compared with non-remitters in TRD (Supplementary Table S10). Among miRNAs regulating CYP2C19, only hsa-let-7d-5p was nominally upregulated in non-remitters compared with remitters in patients with TRD (logFC = 0.27, t = 2.31, *p* = 0.02, adj *p* = 0.46) but not in those without TRD (logFC = 0.03, t = 0.28, *p* = 0.78, adj *p* = 0.99). Finally, CYP2B6 phenotypes were not significantly associated with remission in TRD or non-TRD patients (Table 4 and Supplementary Table S11).

## 4. Discussion

In this cross-sectional, retrospective study, we exploited data produced within the PROMPT project [13] to specifically explore whether genotype-predicted metabolizing phenotypes of CYP genes and differences in their mRNA blood levels and miRNAs predicted to regulate them were involved in TRD. Studies published so far suggest that CYP-based pharmacogenetics of antidepressants may be clinically useful in guiding treatment, though findings have been controversial, and overall their clinical utility remains modest [19,20]. The latter may suggest that the study of other CYP-related molecular features, such as gene expression and epigenetic regulators (miRNAs), which have been mostly neglected so far, might help increase the informativity of pharmacogenetic testing in antidepressant treatment.

In the present study, we showed that CYP2D6 phenotype distribution did not differ significantly between TRD and non-TRD groups, but mRNA expression was significantly upregulated in TRD, while hsa-miR-26b-5p, a microRNA predicted to regulate CYP2D6, was significantly downregulated in TRD. While these findings are somewhat inconsistent, they suggest that mechanisms related to CYP genes other than sequence variation may contribute to modulating the risk of TRD. Nevertheless, because our study did not examine variables potentially involved in phenoconversion, this finding remains a hypothesis that requires further investigation. While limited in numbers, previous studies suggested that changes in CYP gene expression might be associated with different genotype-predicted metabolizing phenotypes. Interestingly, a recent study by Meaddough et al. showed that CYP2D6 gene expression varies widely within genotype-predicted metabolizer groups in human liver tissue, suggesting that transcriptional regulation contributes to phenoconversion and may limit the clinical accuracy of genotype-based pharmacogenetic testing [11].

Regarding psychiatric medications, the importance of differences in CYP mRNA levels has been recently supported by a study showing that peripheral blood CYP1A2 mRNA expression, rather than genotype, was associated with olanzapine concentration in schizophrenia or bipolar disorder patients [21]. While we did not identify a significant association between CYP2D6 metabolizer phenotypes and clinical outcomes, we can speculate that increased gene expression could contribute to an increased elimination rate of the substrate drugs, thus possibly making the “standard” dose inefficacious. Nevertheless, it is important to highlight that our expression study was performed on peripheral blood and that hepatic expression of CYP2D6, as well as information on drug dose and dose adjustments, were not available for the studied sample, and as such, this assumption cannot be confirmed or confuted.

Regarding previous miRNA findings, a study on postmortem brain samples reported that lower levels of miR-26b-5p in the dorsolateral prefrontal cortex were associated with higher depressive symptom scores in participants from two longitudinal clinical–pathological cohort studies of aging (AD-ROS and MAP) [22]. miR-26b-5p was also recently shown to be downregulated in peripheral blood in patients with depression [23] and in extracellular vesicles in patients with depression and childhood trauma [24] compared with controls in two pilot studies on a small number of participants and conducted by the same group. The authors of these studies suggested miR-26b-5p to be a potential mediator of the association between depression and bone health based on the observed correlation between miRNA levels and bone turnover markers [23,24]. Together with the previously published work, our findings may suggest that the pharmacokinetics of psychiatric drugs could also be partly influenced by epigenetically determined mechanisms.

As regards CYP2C19, we observed a significant association between CYP2C19 metabolizer phenotypes and TRD. Non-TRD showed a higher frequency of IM or IM + PM phenotypes compared with TRD patients (Supplementary Table S5). In addition, among patients with TRD, higher frequencies of IM or IM + PM phenotypes were nominally associated with remission (Supplementary Table S9). These results are in accordance with a recent study on 1239 patients with MDD reporting that CYP2C19 PMs had higher rates of response and symptom improvement compared to NMs, but also a higher risk of autonomic and neurological side effects [10]. Accordingly, a meta-analysis of 13 studies on 5843 patients with MDD from European and East Asian ancestry populations reported nominal significance for the association between the CYP2C19 PM phenotype and a higher remission rate [25], while a meta-analysis of 2558 patients with MDD from the GENDEP, STAR ∗D, GenPod, and PGRN-AMPS cohorts reported higher symptom improvement and remission rates in CYP2C19 PMs compared with NMs [26]. However, these results are in contrast with two studies recently conducted in the UK Biobank cohort. Kamp and colleagues found lower rates of self-reported response to selective serotonin reuptake inhibitors in CYP2C19 PMs compared with NMs in 19,516 participants from the UK Biobank [27], while Wong and colleagues analyzed data from 3012 individuals prescribed escitalopram, reporting that CYP2C19 PMs were more likely to switch antidepressants, have side effects following first prescription, and be on escitalopram for a shorter duration compared to NMs [28]. While CYP2C19 mRNA levels could not be analyzed due to low expression of the gene, two CYP2C19-regulating miRNAs were significantly downregulated in patients with TRD compared with non-TRD: let-7d-5p (logFC = −0.31, adj *p* = 0.002) and miR-27a-3p (logFC = −0.38, adj *p* = 0.0008). The latter findings are difficult to interpret in light of the lack of mRNA levels, but at the same time they may support the hypothesis of a possible role of non-genetic factors involved in CYP regulation in response and resistance to psychiatric drugs.

To our knowledge, this study is the first to provide a comprehensive analysis of metabolizer phenotypes, mRNA expression, and regulatory miRNA levels of antidepressant-relevant pharmacogenes in TRD and remission, but its significance and impact should be interpreted cautiously in view of the inconsistency of some findings and the study’s limitations. Firstly, the retrospective nature of the study did not allow us to explore the causative effect of genetic variants or transcript levels in modulating remission and resistance to treatments. Moreover, information on blood drug levels was not available, and likewise details on dosage adjustment during the treatment course. It is also important to note that while the observed changes in cytochrome blood gene expression may suggest that mechanisms other than sequence variants may be involved in modulating TRD and response to antidepressants (and that in some circumstances these could play a role in phenoconversion), these may not necessarily reflect hepatic cytochrome activity. In this regard, a study from Temesvari et al. [29] showed that while for CYP2C19 the activity in leukocytes reflects the hepatic expression and activity, this was not true for CYP2D6. Moreover, data on blood mRNA levels were not available. As such, we can conclude that the lack of hepatocyte-derived findings in our study limits the possibility to draw direct conclusions about the contribution of CYP gene expression and its epigenetic regulation to the hepatic metabolism of CYP substrates. Another important limitation is represented by the relatively small sample, which limited the number of participants with extreme phenotypes that could be included in the analyses focused on remission. In addition, all participants were of European origin, thus potentially limiting the application of these results to other populations.

## 5. Conclusions

By extending the analyses beyond genetically inferred CYP-metabolizing phenotypes, our pharmacogenetic study suggests that gene expression and regulating miRNAs might play a role in efficacy of antidepressants and in TRD, although the limitations described above highlight the need for further investigations involving larger samples, therapeutic drug monitoring and drug levels, and possibly studies on hepatocytes. Therefore, this study should be interpreted as exploratory and the findings as suggestive of the potential advantages of an integrated approach to explore the pharmacogenetics of antidepressants and its involvement in TRD.

## Figures and Tables

**Table 1:** Sociodemographic and clinical features of the sample.

Sociodemographic and Clinical Features	Non-TRD N = 150	TRD N = 150	*p*-Value *
Age in years, mean (SD)	48.7 (15.9)	55.8 (11.19)	<0.001
Females, *n* (% F)	109 (72.7)	106 (70.7)	0.798
Smokers, *n* (%) ^1^	53 (40.2)	54 (37.5)	0.711
Body mass index (BMI), mean (SD) ^2^	24.3 (4.9)	25.2 (4.9)	0.097
Age of onset in years, mean (SD) ^3^	39.1 (14.9)	33.4 (13.7)	<0.001
MADRS score at recruitment (baseline), mean (SD)	26.1 (5.8)	31.9 (7.3)	<0.001
Comorbidity with anxiety disorders, *n* (%)	53 (35.3)	38 (25.3)	0.078

* Non-TRD vs. TRD patients, tested with Mann–Whitney U test or Pearson’s chi-squared test; ^1^ available for 149 non-TRD and 150 TRD; ^2^ available for 132 non-TRD and 144 TRD; ^3^ available for 126 non-TRD and 140 TRD. Abbreviations: F, female; n, number; SD, standard deviation.

**Table 2:** Associations between pharmacogene metabolizer phenotypes and TRD or remission.

Pharmacogene Metabolizer Phenotype	Non-TRD N = 150	TRD N = 150	χ^2^	*p*-Value	Non-TRD Remitters N = 75	TRD Non-Remitters N = 57	χ^2^	*p*-Value
**CYP2D6 phenotypes (%) ^1^**	PMs = 4.7 IMs = 39.6 NMs = 51.7 UR = 4.0	PMs = 4.1 IMs = 35.1 NMs = 58.1 UMs = 2.7	1.41	0.703	PMs = 4.0 IMs = 40.0 NMs = 53.3 UR = 2.7	PMs = 3.5 IMs = 31.6 NMs = 64.9 UR = 0.0	2.92	0.405
**CYP2C19 phenotypes (%) ^2^**	PMs = 4.7 IMs = 32.0 NMs = 33.3 RMs = 27.3 UMs = 2.7	PMs = 2.0 IMs = 20.1 NMs = 43.6 RMs = 28.2 UMs = 6.0	9.64	**0.047**	PMs = 6.7 IMs = 44.0 NMs = 25.3 RMs = 22.7 UMs = 1.3	PMs = 5.3 IMs = 19.3 NMs = 45.6 RMs = 21.1 UMs = 8.8	13.92	**0.008**
**CYP2B6 phenotypes (%) ^3^**	PMs = 10.7 IMs = 36.7 NMs = 51.3 RMs = 1.3	PMs = 6.8 IMs = 47.6 NMs = 42.2 RMs = 3.4	6.06	0.109	PMs = 10.7 IMs = 37.3 NMs = 52.0 RMs = 0.0	PMs = 1.8 IMs = 52.7 NMs = 40.0 RMs = 5.5	10.37	**0.016**

^1^ Available for 149 non-TRD and 148 TRD; ^2^ available for 150 non-TRD and 149 TRD; ^3^ available for 150 non-TRD and 147 TRD and for 74 non-TRD remitters and 55 TRD non-remitters. Results significant after multiple testing correction are reported in bold. Abbreviations: IMs, intermediate metabolizers; NMs, normal metabolizers; PMs, poor metabolizers; RMs, rapid metabolizers; UMs, ultrarapid metabolizers.

**Table 3:** Associations between pharmacogene mRNA, miRNAs regulating pharmacogenes, and TRD or remission.

Pharmacogene or miRNA	logFC	t	*p*-Value	adj *p*-Value *
**Association with TRD vs. non-TRD**				
CYP2D6	**0.35**	**4.54**	**8.3 × 10^−6^**	**0.0002**
*miRNAs regulating CYP2D6*				
hsa-miR-26b-5p	**−0.62**	**−5.27**	**2.6 × 10^−7^**	**2.5 × 10^−5^**
*miRNAs regulating CYP2C19*				
hsa-miR-30d-3p	−0.11	−1.12	0.26	0.51
hsa-let-7d-5p	**−0.31**	**−4.18**	**3.9 × 10^−5^**	**0.002**
hsa-miR-139-5p	−0.23	−2.46	0.01	0.10
hsa-miR-210-3p	0.20	2.55	0.01	0.09
hsa-miR-27a-3p	**−0.38**	**−4.35**	**1.9 × 10^−5^**	**0.0008**
hsa-miR-423-5p	0.08	1.39	0.17	0.40
**Association with non-remission in TRD vs. remission in non-TRD**				
CYP2D6	0.29	2.47	**0.01**	0.11
*miRNAs regulating CYP2D6*				
hsa-miR-26b-5p	−0.44	−2.33	**0.02**	0.21
*miRNAs regulating CYP2C19*				
hsa-miR-30d-3p	−0.01	−0.05	0.96	0.99
hsa-let-7d-5p	−0.19	−1.68	0.09	0.42
hsa-miR-139-5p	−0.23	−1.64	0.10	0.44
hsa-miR-210-3p	0.19	1.64	0.10	0.44
hsa-miR-27a-3p	−0.44	−3.14	**0.002**	0.07
hsa-miR-423-5p	0.00	0.04	0.97	0.99

* Adjusted *p*-value based on the original RNA [14] and miRNA sequencing studies (Sirés et al. [15]). Results significant after multiple testing correction are reported in bold.

**Table 4:** Associations between pharmacogene metabolizer phenotypes and remission.

	Non-TRD				TRD			
Pharmacogene Metabolizer Phenotype	Remitters N = 75	Non-Remitters N = 40	χ^2^	*p*-Value	Remitters N = 55	Non-Remitters N = 57	χ^2^	*p*-Value
**CYP2D6 phenotypes (%) ^1^**	PMs = 4.0 IMs = 40.0 NMs = 53.3 UR = 2.7	PMs = 2.5 IMs = 50.0 NMs = 45.0 UR = 2.5	1.38	0.709	PMs = 3.7 IMs = 35.2 NMs = 53.7 UR = 7.4	PMs = 3.5 IMs = 31.6 NMs = 64.9 UR = 0.0	4.92	0.178
**CYP2C19 phenotypes (%)**	PMs = 6.7 IMs = 44.0 NMs = 25.3 RMs = 22.7 UMs = 1.3	PMs = 2.4 IMs = 19.5 NMs = 34.1 RMs = 41.5 UMs = 2.4	10.66	0.031	PMs = 0.0 IMs = 14.5 NMs = 43.6 RMs = 36.4 UMs = 5.5	PMs = 5.3 IMs = 19.3 NMs = 45.6 RMs = 21.1 UMs = 8.8	6.02	0.198
**CYP2B6 phenotypes (%) ^2^**	PMs = 10.7 IMs = 37.3 NMs = 52.0 RMs = 0.0	PMs = 14.6 IMs = 29.3 NMs = 51.2 RMs = 4.9	4.90	0.179	PMs = 3.6 IMs = 50.9 NMs = 43.6 RMs = 1.8	PMs = 1.8 IMs = 52.7 NMs = 40.0 RMs = 5.5	1.44	0.697

^1^ Available for 39 non-TRD remitters, 75 TRD non remitters, 54 TRD remitters, and 57 TRD non remitters; ^2^ available for 40 non-TRD remitters, 75 TRD non remitters, 55 TRD remitters and 55 TRD non remitters. Abbreviations: IMs, intermediate metabolizers; NMs, normal metabolizers; PMs, poor metabolizers; RMs, rapid metabolizers; UMs, ultrarapid metabolizers.

## Data Availability

The datasets used and/or analyzed during the current study are available from the corresponding author on reasonable request.

## Supplementary Materials

The following supporting information can be downloaded at https://www.mdpi.com/article/10.3390/medicina62050965/s1. Supplementary Table S1. Socio-demographic and clinical features of the patients characterized as remitted after 8 weeks of treatment on the basis of MADRS assessment and stratified based on TRD. Supplementary Table S2. Haplotype-inferred phenotype. Supplementary Table S3. Association between pharnacogene metabolizer phenotypes and clinical variables. Supplementary Table S4. Association between CYP2D6 phenotypes and TRD or extreme phenotypes of response. Supplementary Table S5. Association between CYP2D6 metabolizer phenotypes and TRD or extreme phenotypes of response in patients treated with antidepressants with CYP2D6 clinical annotations. Supplementary Table S6. Association between CYP2C19 phenotypes and TRD or extreme phenotypes of response. Supplementary Table S7. Association between CYP2C19 metabolizer phenotypes and TRD or extreme phenotypes of response in patients treated with antidepressants with CYP2C19 clinical annotations. Supplementary Table S8. Association between CYP2B6 phenotypes and TRD or extreme phenotypes of response. Supplementary Table S9. Association between CYP2D6 phenotypes and remission in patients stratified for TRD. Supplementary Table S10. Association between CYP2C19 phenotypes and remission in patients stratified for TRD. Supplementary Table S11. Association between CYP2B6 phenotypes and remission in patients stratified for TRD.

## Abbreviations

The following abbreviations are used in this manuscript.

TRD	treatment-resistant depression
MDD	major depressive disorder
mRNA	messenger RNA
miRNA	microRNA
NMs	normal metabolizers
IMs	intermediate metabolizers
PMs	poor metabolizers
UMs	ultrarapid metabolizers
CYP	cytochrome
DSM-IV	Diagnostic and Statistical Manual of Mental Disorders—Fourth Edition
LogFC	logarithm of fold change
Adj *p*	adjusted *p* value
OR	odds ratio
AD-ROS -MAP	Religious Orders Study (ROS)/Rush Memory and Aging Project (MAP)
GENDEP	Genome-Based Therapeutic Drugs for Depression
STAR*D	Sequenced Treatment Alternatives to Relieve Depression
GenPod	GENetic and clinical Predictors Of treatment response in Depression
PGRN-AMPS	Pharmacogenomic Research Network Antidepressant Medication Phamacogenomic Study
PROMPT	Toward PrecisiOn Medicine for the Prediction of Treatment response in major depressive disorder through stratification of combined clinical and -omics signatures
OCD	obsessive compulsive disorder
PTSD	posttraumatic stress disorder
MADRS	Montgomery–Åsberg Depression Rating Scale
BMI	body mass index
CNV	copy number variant
CPIC	Clinical Pharmacogenetics Implementation Consortium
SNP	single-nucleotide polymorphism
